# The Peripandemic Impact of the First Wave of the COVID-19 Pandemic on Management and Prognosis of ST-Segment Elevation Myocardial Infarction in China

**DOI:** 10.3390/jcm11247290

**Published:** 2022-12-08

**Authors:** Hongbo Yang, Lingfeng Luo, Jiatian Cao, Yanan Song, Xueyi Weng, Feng Zhang, Xiaofeng Zhou, Yong Huo, Juying Qian, Yan Zheng, Zheyong Huang, Junbo Ge

**Affiliations:** 1 Department of Cardiology, Zhongshan Hospital, Fudan University, Shanghai Institute of Cardiovascular Diseases, National Clinical Research Center for Interventional Medicine, Shanghai 200032, China; 2 Department of Occupational and Environmental Health, School of Public Health, Medical College of Soochow University, Suzhou 215123, China; 3 Human Phenome Institute, Fudan University, Shanghai 200433, China; 4 Department of Cardiology, Peking University First Hospital, Beijing 100044, China; 5 Department of Cardiology, Zhongshan Hospital, Fudan University, Shanghai 200437, China; 6 State Key Laboratory of Genetic Engineering, School of Life Sciences and Human Phenome Institute, Fudan University, Shanghai 200437, China

**Keywords:** COVID-19, STEMI, residual impact

## Abstract

Background: Rapid reperfusion of ST-segment elevation myocardial infarction (STEMI) has been challenging during the coronavirus disease 2019 (COVID-19) outbreak. Whether and to what degree there will be a residual impact when the COVID-19 pandemic has passed is unclear. Methods: This nationwide retrospective study was based on electronic records of STEMI patients registered in the Chinese Cardiovascular Association Database. Results: We analyzed 141,375 STEMI patients (including 4871 patients in Hubei province, where 80% of COVID-19 cases in China occurred in 2019–2020) during the pre-outbreak (23 October 2019–22 January 2020), outbreak (23 January 2020–22 April 2020), and post-outbreak (23 April 2020–22 July 2020) periods. In the post-outbreak period in Hubei province, the increased in-hospital mortality dropped to become insignificant (adjusted odds ratio compared to the pre-outbreak level (aOR) 1.40, [95% confidential interval (CI): 0.97–2.03]) and was lower than that in the outbreak period (1.62 [1.09–2.41]). The decreased odds of primary percutaneous coronary intervention (PCI) (0.73 [0.55–0.96]) and timely reperfusion (0.74 [0.62–0.88]) persisted, although they were substantially improved compared to the outbreak period (aOR of primary PCI: 0.23 [0.18–0.30] and timely reperfusion: 0.43 [0.35–0.53]). The residual impact of COVID-19 on STEMI in the post-outbreak period in non-Hubei provinces was insignificant. Conclusions: Residual pandemic impacts on STEMI management persisted after the first wave of the COVID-19 outbreak in Hubei province, the earliest and hardest hit area in China.

## 1. Introduction

Coronary artery disease affects more than seven million individuals worldwide each year, and its incidence has been rapidly increased in China during the past decade [1,2]. Acute myocardial infarction is the most severe manifestation of coronary artery disease [1]. As the most common subclass of acute myocardial infarction, ST-segment elevation myocardial infarction (STEMI) is time sensitive, and any delay by patients, emergency medical services (EMS), emergency departments, or catheterization laboratories would severely hamper effective reperfusion, and cause a worse prognosis [3,4]. High numbers of cases and deaths have occurred during the past two years of the coronavirus disease 2019 (COVID-19) pandemic [5], which has caused increased cardiovascular mortality directly and indirectly [6]. Direct effects include the cardiac injury caused by the coronavirus infection and myocardial infarction triggered by excessive vascular inflammation, endothelial dysfunction, platelet activation, and coagulation/fibrinolysis disturbances [7]. Indirect effects are mainly due to the delay or avoidance of hospital visits and the shortages of medical staff [3,8]. It was reported that 8–12% of COVID-19 infections in Italy and Spain were among healthcare workers, raising the concern of nosocomial infection [9]. The China Chest Pain Center Executive Committee recommended prioritizing thrombolysis for STEMI patients with unconfirmed COVID-19 to balance the need for timely reperfusion and infection control [10] which, though it prevented nosocomial infection, resulted in fewer primary percutaneous coronary interventions (PCI), and consequently an increase in in-hospital mortality [8].

It is unknown whether these adverse impacts persisted after the containment of COVID-19. In the first year of the COVID-19 pandemic, several countries, such as China, Singapore, Australia, and New Zealand [11], aimed to completely stop community transmission. However, few studies assessed the residual impact due to loss of control over the COVID-19 pandemic. One study indicated an increase in admissions for acute myocardial infarction (AMI) after the removal of the national lockdown for COVID-19 in Italy [12]; another study reported AMI rates recovered to those of the pre-epidemic era in 16 to 19 weeks throughout northern California during the most recent COVID-19 surges in the United States [13]. However, removal of lockdown with many COVID-19 cases daily in these countries is different from our study, where we have been without any locally acquired COVID-19 cases for several months. China is ideal for exploring this question because China eliminated local infection and contained the epidemic of the original wild-type virus within three months through strict mask wearing, travel restrictions, etc. [14,15,16]. Timelines of the COVID-19 pandemic and containment in China were clear: the outbreak period started on 23 January 2020 (when Wuhan, the capital city of Hubei province, launched a lockdown) and ended on 22 April 2020 (two weeks after the lockdown was removed). Accordingly, we defined the pre-outbreak period (13 October 2019, to 22 January 2020) and the post-outbreak period (23 April to 22 July 2020). Because Hubei province was the earliest and hardest hit area in China [17,18], with about 80% of the first wave of COVID-19 cases restricted to the Hubei province during the COVID-19 pandemic [19]. Hence, impacts on management and prognosis were analyzed in STEMI admissions in Hubei and non-Hubei provinces.

In this study, by using the Chinese Cardiovascular Association (CCA) Database–Chest Pain Centers (a nationwide database) [20], we aimed to investigate the influence of the COVID-19 pandemic on STEMI patients, with a particular focus on the residual impact during the post-outbreak period in China.

## 2. Materials and Methods

### 2.1. Study Design and Population

The current study extracted data from the ongoing, permanent, prospective CCA Database–Chest Pain Centers established in 2015. All participating chest pain centers are required to register and report demographic information, diagnosis, treatment, and timelines of all patients presenting with acute chest pain. Chest pain centers employ trained personnel to collect detailed information on patient and hospital characteristics, coronary angiographic and procedural findings, and outcomes by using standardized data elements.

We included data collected on all STEMI patients, diagnosed according to the fourth universal definition of myocardial infarction [21], who were admitted to all chest pain centers in China during the 9-month study period. The following variables were extracted: age, sex, documented cardiovascular risk factors (e.g., hypertension, diabetes, dyslipidemia, and smoking habit), self-reported medical history, clinical characteristics at admission, pattern of patient arrival, and loading drug use at the emergency department and reperfusion strategy. Because about 80% of the first wave of COVID-19 cases in China were concentrated in Hubei province, we explored the impact of the COVID-19 pandemic on STEMI patients in Hubei and non-Hubei provinces, respectively.

### 2.2. Outcomes

The primary outcome was in-hospital mortality, which was documented in the electronic medical record. The secondary outcomes were evaluated based on three aspects: (1) patient delay: the time from symptom onset to first medical contact (FMC); (2) system delay (primary PCI) [9]: for primary PCI, the time from FMC to wire crossing, where system delay (primary PCI) ≤120 min indicated timely reperfusion, and system delay (fibrinolysis): the time from FMC to needle (the beginning of thrombolysis), where system delay (fibrinolysis) ≤30 min indicated timely reperfusion; (3) reperfusion strategies: proportion of primary PCI or thrombolysis.

### 2.3. Association between COVID-19 Prevalence and Changes in STEMI Management and Prognosis in China

In this study, we aimed to estimate the association between COVID-19 prevalence and changes in STEMI management and prognosis by using an ecological approach to analyze data from the 31 provinces in China. We collected cumulative confirmed cases of COVID-19 from 23 January to 22 April and from 23 January to 22 July 2020, from the official website of the National Health Commission of China. We obtained the total population size (aged 15 or older) of 31 provinces in China from the official website of the National Bureau of Statistics [22]. The prevalence of COVID-19 from 23 January to 22 April and from 23 January to 22 July 2020, was calculated as follows:

$$\mathrm{Prevalence}\;\mathrm{of}\;\mathrm{COVID}\text{-}19=\frac{\mathrm{Cumulative}\;\mathrm{COVID}\text{-}19\;\mathrm{cases}}{\mathrm{Total}\;\mathrm{Population}\;\mathrm{Size}}\;*10000.$$

In this analysis, we investigated the association between COVID-19 prevalence and related indicators, including (1) changes in STEMI admissions, (2) changes in patient delay and system delay, and (3) changes in the proportion of primary PCI, thrombolysis, timely reperfusion, and in-hospital mortality. Multivariate linear regression analyses were conducted, adjusting for the confounding factors of economy, population, and medical level, i.e., the average age of residence in each province, the number of chest pain centers in each province, gross domestic product (GDP) (trillion RMB Yuan in 2019) of the province, spatial distances (100 km) from the geographic center of individual provinces to Hubei estimated by using the software Arc GIS (Environment System Research Institute, State of California, American, v 10.0) with geographic data) [23], and population density (100 persons/km^2^, calculated by dividing the population by the geographic area in each province). Population data, average age, and GDP for the 31 provinces were obtained from the 2019 National Bureau of Statistics of China, and geographic area data were obtained from the website [22].

### 2.4. Statistical Analysis

Means and standard deviations were used as descriptive statistics for continuous variables if they followed a normal distribution; otherwise, median and interquartile range [IQR] were applied. Between-group comparisons of these continuous variables were conducted by using the ANOVA test, followed by multiple comparisons using the least significant difference (LSD) test or Kruskal–Wallis nonparametric test, with Dunn’s multiple comparison. For categorical variables, the proportion was used to show prevalence in our study population, and Chi-square tests and Fisher’s exact test were applied to compare between groups. Multivariable-adjusted (age, gender, hypertension, diabetes, coronary heart disease, history of chronic heart failure, stroke, heart rate, systolic blood pressure, and time of symptom onset to first medical contact) logistic regression models were developed to estimate the influence of the COVID-19 outbreak. Multivariate linear regression analysis was used to explore the association between COVID-19 prevalence and changes in STEMI management in the outbreak and post-outbreak periods. All comparisons were two-sided, with statistical significance defined as *p* < 0·05. Statistical analysis was conducted with R software (R Foundation for Statistical Computing, Vienna, Austria, v. 4.1.2).

## 3. Results

The characteristics of the study population are presented in Figure S1. According to the CCA Database–Chest Pain Centers, 1,418,667 patients were registered in the study period, and 174,157 of them were diagnosed as STEMI during the study period. We excluded 9166 patients missing values for symptom onset time or sex, and 23,616 patients receiving reperfusion for over 24 h since symptom onset. Thus, 141,375 patients (47,560 from the pre-outbreak period, 45,261 from the outbreak period, and 48,554 from the post-outbreak period) were enrolled in the final analysis and classified into different groups. We observed a 4.8% reduction in admissions, 0.2 h patient delay, 2.3% decrease in primary PCI, 2.6% increase in thrombolysis, and 0.5% increase in in-hospital mortality for STEMI patients during the outbreak period compared with the pre-outbreak period (*p* < 0.0001) (Tables S1 and S2). Specifically, admissions for STEMI showed a 34.5% reduction in the outbreak period (*n* = 1253) and a 10.9% reduction in the post-outbreak period (*n* = 1705) compared with the pre-outbreak period (*n* = 1913) in Hubei. At the same time, it remained stable in non-Hubei chest pain centers (Table 1). Detailed distributions of demographic characteristics, risk factors of cardiovascular diseases, comorbidities, and left ventricular ejection fraction are presented in Table 1.

### 3.1. Patient Delay and Management in the Emergency Department

During the outbreak period in Hubei centers, patient delay, presented as median patient delay time, increased from 2.1 h to 2.8 h (*p* < 0.0001), and it returned to 2.2 h in the post-outbreak period (*p* = 0.10) (Table 2). In a detailed analysis of patient delay month by month, we observed extra prolongation of 0.75 h during the outbreak period in Hubei, and it dropped in the first month of the post-outbreak period. Patient delay in the last month recovered to the level of the first month of the study period in both the Hubei and non-Hubei provinces (Figure S2a). The proportions of loading drug use, of increased clopidogrel (30.6%) and decreased ticagrelor (60.5%), in the outbreak period in Hubei province returned to 24.8% and 66.0% in the post-outbreak period, respectively. Patient delay was prolonged by only 0.2 h, and loading drug use varied by less than 5% during the study period in STEMI patients admitted to non-Hubei chest pain centers (Table 2).

### 3.2. System Delay and Reperfusion Strategy

System delay worsened in primary PCI and thrombolysis in Hubei during the outbreak period, indicated by an extra 36 min of system delay (primary PCI) time (*p* < 0.0001) and an additional 12.5 min of system delay (fibrinolysis) time (*p* = 0.001). Therefore, only 41.7% of patients received timely reperfusion during the outbreak period, and it partly recovered to 55.8% (*p* < 0·0001) during the post-outbreak period. Among the STEMI patients admitted in non-Hubei provinces, system delay (primary PCI) was slightly influenced, as indicated by a 6-min prolongation of system delay (primary PCI) time (*p* < 0.0001) in the outbreak period. However, this influence was insignificant in the post-outbreak period (Table 2). Compared with the reduced proportion (57.0%) of STEMI patients undergoing primary PCI in Hubei chest pain centers in the outbreak period, it increased sharply to 73.7% (*p* < 0·0001) in the post-outbreak period. The proportion of thrombolysis dropped dramatically from 23.9% to 10.9% (*p* < 0.0001) during the post-outbreak period, similar to the 10.4% (*p* = 0.36) in the pre-outbreak period. Changes in proportions of primary PCI and thrombolysis were less than 3% in non-Hubei Chest Pain Centers.

**Figure 1:**
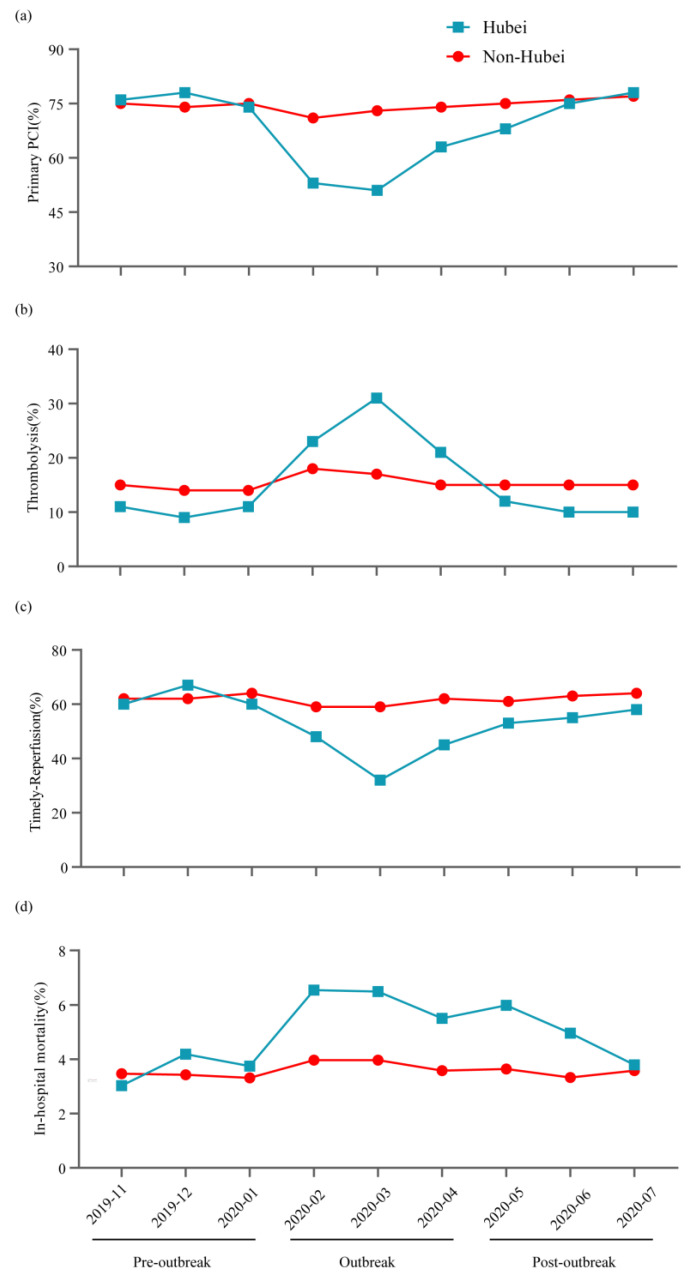
Changes of reperfusion and mortality. Percentages of STEMI patients receiving primary percutaneous coronary intervention (PCI) (**a**), thrombolysis (**b**), timely reperfusion (system delay (primary PCI) ≤120 min or system delay (fibrinolysis) ≤30 min) (**c**), and patterns of in-hospital mortality (**d**).

A detailed analysis found that the most severely affected time was during the mid-term of the outbreak period in Hubei, during which the minimum proportion primary PCI (Figure 1a), peak proportion of thrombolysis (Figure 1b), lowest percentage of timely reperfusion (Figure 1c), and the longest median system delay (primary PCI) and median system delay (fibrinolysis) of 155 min and 67 min (Figure S2b,c) were recorded.

### 3.3. In-Hospital Mortality

Compared to the outbreak period in Hubei, the in-hospital mortality during the post-outbreak period significantly dropped from 6.1% during the outbreak period to 4.9% (*p* = 0.18) in the post-outbreak period (Table 2). Although in-hospital mortality increased by 0.4% (*p* < 0.0001) in non-Hubei provinces during the outbreak period, it returned to 3.5% (*p* = 0.35) in the post-outbreak period.

Detailed changes in in-hospital mortality are illustrated in Figure 1d. In the pre-outbreak period, similar in-hospital mortality was observed in Hubei and non-Hubei provinces. In-hospital mortality peaked at 6.6% in the first month of the COVID-19 outbreak in Hubei, followed by a progressive decline. In-hospital mortality decreased to 3.8% in the last month of the study period in Hubei, close to the level in the pre-outbreak period (3.7%). In-hospital mortality fluctuated within a narrow range in non-Hubei provinces in the study period.

### 3.4. Multivariable Logistic Regression Analysis

**Figure 2:**
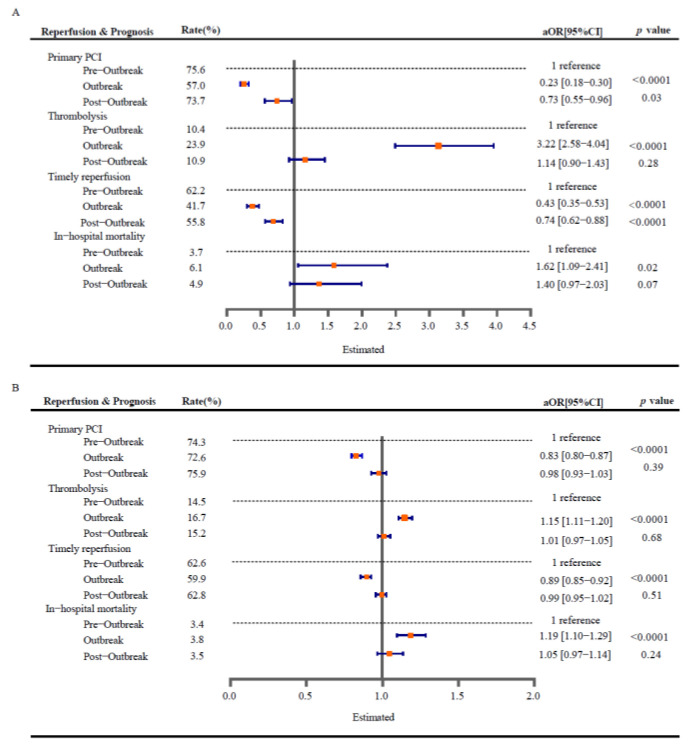
Impact of COVID-19 periods on changes in management and prognosis of STEMI. Regression results of reperfusion patterns and in-hospital outcomes in chest pain centers in Hubei (**A**) and non-Hubei provinces (**B**), adjusted for age, sex, hypertension, diabetes, coronary heart disease, history of chronic heart failure, stroke, heart rate, systolic blood pressure, and time of symptom to first medical contact. aOR, adjusted odds ratio. CI, confidential interval.

Results from the multivariable logistic regression analysis showed that the increased in-hospital mortality during the outbreak period (adjusted odds ratio (aOR) 1.62, [95% confidential interval (CI): 1.09–2.41]) reduced during the post-outbreak period (aOR 1.40, [95% CI: 0.97–2.03]) in Hubei province. Proportions of primary PCI and timely reperfusion were largely decreased in the outbreak period (aOR 0.23, [95% CI: 0.18–0.30] and 0.43, [95% CI: 0.35–0.53]), and partly recovered in the post-outbreak period (aOR 0.73, [95% CI: 0.55–0.96] and 0.74, [95% CI: 0.62–0.88]). Proportions of thrombolysis increased sharply in the outbreak period (aOR 3.22, [95% CI: 2.58–4.04]) and returned to previous levels in the post-outbreak period. Multivariable logistic regression analyses in non-Hubei provinces showed a similar but weakened trend (aOR for mortality 1.19, [95% CI: 1.10–1.29]), and the detrimental effects vanished during the post-outbreak period (Figure 2).

### 3.5. Association between COVID-19 Prevalence and Changes in STEMI Management and Prognosis in China

The prevalence of COVID-19 during the outbreak period in China was associated with decreases in hospital admissions, proportions of primary PCI, and timely perfusion of STEMI patients, and increases in patient delay of patient delay time (Table 3). Each COVID-19 case per 10,000 residents during the outbreak period was associated with a 2.75% reduction in STEMI hospital admissions, a 3 min reduction in patient delay time, and a 1.31% and 1.39% decrease in the proportion of primary PCI and timely perfusion compared to the pre-outbreak period. The thrombolysis and in-hospital mortality showed an increasing trend but did not reach statistical significance (*p* = 0.07 for thrombolysis, *p* = 0.10 for mortality). No significant association was observed between the nationwide prevalence of COVID-19 and changes in STEMI management and prognosis during the post-outbreak period (Table 3).

## 4. Discussion

By utilizing a large sample size of registered STEMI patients from the CCA Database–Chest Pain Centers, our study reported that among STEMI patients in Hubei province during the post-outbreak period, the in-hospital mortality had nearly returned to the pre-outbreak level, but the residual impacts on patient delay, system delay, and reperfusion strategies still persisted. In addition, the prevalence of COVID-19 during the outbreak period significantly impacted the admissions and management for STEMI patients, and such impacts were weakened during the post-outbreak period.

During the post-outbreak period, we observed a similar level of in-hospital mortality as that in the pre-outbreak period in China. Though studies focused on post-outbreak impacts are limited, many observations of the outbreak impacts were reported. The higher percentage of in-hospitality mortality of STEMI during the outbreak in China was consistent with findings in Italy, France, and other countries [24,25,26], but in contrast to those in the United Kingdom, the United States, and Sweden [27,28,29]. One possible reason for the results regarding mortality in the outbreak period is the different strategies and attitudes towards the pandemic. In countries with a stringent lockdown strategy and “zero COVID” strategy, the EMS and operation date in STEMI patients were restructured and postponed in the majority of hospitals [24,30]. This approach to public health and technology allowed aggressive containment to succeed [15], whereas disadvantages were obvious in some aspects, such as worsening fear of contagion and aggravating a lack of medical service for non-COVID-19 acute diseases. Although the adverse impact was nearly eliminated in China three months after the outbreak, the ongoing impacts on fear of resurgence and hospital avoidance behaviors still existed in Hubei province, which may have been reflected in decreased admissions in Hubei but increased admissions in other provinces. The recurrent short-term local epidemic of COVID-19 in China suggested that this concern was reasonable. When the COVID-19 epidemic is controlled, our findings suggested that the health system in severely impacted areas should adopt public health campaigns to reassure patients about the timely treatment of seeking emergency care when needed.

During the outbreak period, our results were consistent with other study findings, with varying degrees of increase in patient delay and system delay [4,9]. In our study, the COVID-19 pandemic in the post-outbreak period still had a significant impact on patient delay and system delay. The persistent impacts originated from the stringent strategies of lockdown and zero COVID [24,30], and in-hospital nucleic acid amplification testing may have been a factor in system delay in STEMI patients in China. The patient delay was still prolonged in non-Hubei provinces in the post-outbreak period, which may have been related to increased hospital visits.

We observed that residual effects on reperfusion strategies in STEMI patients still existed in Hubei province after the COVID-19 pandemic, while they were reduced in other provinces in China. Patients with or without COVID-19 during the pandemic were less likely to undergo invasive coronary procedures [9,31,32]. Regarding the reperfusion strategies during the COVID-19 pandemic, classification and management of STEMI patients according to the risk of COVID-19 infection and severity of STEMI in China may have been better than simply encouraging thrombolysis or primary PCI [4,11,33]. Primary PCI should be recommended and quickly adopted in low-risk regions, especially for severe STEMI patients. Italy and Spain had a high infection rate of COVID-19 among medical staff [10,34,35], and one study reported that 6.3% of STEMI patients were infected with COVID-19 in Spain [30]. Therefore, a more reasonable triage method should be established to balance optimal treatment for STEMI patients and protect medical workers and patients against infection risk based on the severity of locally acquired cases. Our study suggested that classified STEMI management is necessary because indiscriminative thrombolysis may lead to excessive in-hospital mortality.

A drastic reduction in STEMI admissions was observed in Hubei province during the COVID-19 outbreak period, and the residual impact was sustained in the post-outbreak period. Decreased admissions during the lockdown were reported in many high-risk countries such as Italy, Spain, the United States, France, and England [24,30,36,37,38], which is quite different from previous external triggers, such as natural disasters, wars, sports events, cold weather, or influenza pandemics inducing MI [39,40]. As the impact of climate on the incidence of AMI has been documented [41,42], we also extracted the data from corresponding months from 2018 to 2019 (23 October 2018, to 22 January 2019; 23 January 2019, to 22 April 2019; and 23 April 2019, to 22 July 2019) to exclude the influence of seasonality. Compared to the nadir of STEMI admission in the outbreak period in 2020, we observed peak admission in the corresponding time in 2019 (Figure S3). Therefore, the reduced STEMI cases during the outbreak was due to the impact of COVID-19 rather than the seasonality of different study periods. On the contrary, the rebound of AMI was observed in a few countries, even during uncontrolled COVID-19 pandemic periods [43,44,45,46]. A relatively strict policy of staying at home and patients’ fear of infection [3,47] may have affected STEMI treatment in China, while some campaigns were adopted to encourage patients with symptoms or signs of AMI to seek immediate medical attention, even amid the pandemic, in several countries [48,49,50]. Staying at home and self-quarantining should distinguish nonessential activity from necessary medical care [51]. As the pandemic continues, it will be imperative for healthcare providers to emphasize the importance of timely care for patients with STEMI [46].

This study had some limitations. The CCA Database–Chest Pain Centers collected data on patients admitted to chest pain centers, but not all patients in China, and the STEMI patients recorded in the database may have differed from other STEMI patients. Despite the possibility of selection bias in the patients admitted to chest pain centers, a decrease in the percentage of conservative therapy, an increase in primary PCI, and a decrease in in-hospital mortality when compared to the China–PEACE research [52] showed that these facilities have been raising the standard of care in China; however, COVID-19 interfered with the impact size to varying degrees. The Killip class, an important predictor of mortality, was not collected during the study period, which may have influenced the findings. Due to the lack of data on COVID-19 nucleic acid diagnostic tests and long-COVID-19, the potential impact of COVID-19 infections and post-acute COVID-19 syndrome on incidence and outcomes for STEMI patients cannot be assessed in the current study. A lack of information about healthcare labor shortages in China may have caused biases in the estimate of administrative times. A lack of peak troponin concentrations may have hampered adjustment of the confounding effect of infarct size. In addition, we only investigated the original wild-type virus; the impact of other types, such as the Delta and Omicron variants, may be quite different, and further studies are warranted in the future.

## 5. Conclusions

After the containment of the first wave of the COVID-19 pandemic, though in-hospital mortality nearly returned to the pre-outbreak level, residual impacts on reperfusion strategies persisted among STEMI patients in Hubei province, where aggressive control strategies were used. Better strategies are needed to ameliorate this adverse influence on STEMI care when striving against contagion with aggressive measures in future.

## Figures and Tables

**Table 1:** Baseline characteristics.

	Hubei			Non-Hubei		
	Pre-Outbreak (*n* = 1913)	Outbreak (*n* = 1253)	Post-Outbreak (*n* = 1705)	Pre-Outbreak (*n* = 45,647)	Outbreak (*n* = 44,008)	Post-Outbreak (*n* = 46,849)
Demographic						
Age, years old	62.3 ± 12.3	60.4 ± 12.3 *	61.8 ± 12.2 #	62.3 ± 13.0	61.9 ± 12.8 *	61.8 ± 13.0 *
Women (%)	454 (23.7)	264 (21.1)	382 (22.4)	10,838 (23.7)	9750 (22.2) *	10,692 (22.8) *#
CVD ^1^ Risk factors						
Hypertension (%)	780 (40.8)	515 (41.1)	810 (47.5)	17,450 (38.2)	16,968 (38.6)	19,051 (40.7)
Diabetes (%)	290 (15.2)	182 (14.5)	361 (21.2) *#	6446 (14.1)	7329 (16.7) *	8472 (18.1) *
Dyslipidemia (%)	301 (15.7)	207 (16.5)	296 (17.4)	6515 (14.3)	7202 (16.4) *	8456 (18.0) *#
Smoking (%)	598 (31.3)	412 (32.9)	600 (35.2)	12,330 (27.0)	13,452 (30.6) *	15,531 (33.2) *
Family history of CAD ^2^ (%)	55 (2.9)	32 (2.6)	41 (2.4)	1213 (2.7)	1243 (2.8)	1335 (2.8)
Number of risk factors						
None (%)	327 (17.1)	259 (20.7)	338 (19.8) #	7729 (16.9)	7990 (18.2) *	9045 (19.3) *
1 (%)	538 (28.1)	390 (31.1)	556 (32.6)	11,509 (25.2)	12,449 (28.3)	13,962 (29.8)
2 (%)	351 (18.3)	237 (18.9)	318 (18.7)	6745 (14.8)	7369 (16.7)	8521 (18.2)
≥3 (%)	286 (15.0)	184 (14.7)	357 (20.9)	6353 (13.9)	7213 (16.4)	8291 (17.7)
Medical history						
CAD (%)	581 (30.4)	543 (43.3) *	745 (43.7) *	12,504 (27.4) *	13,643 (31.0)	16,215 (34.6) *#
Chronic heart failure (%)	114 (6.0)	91 (7.3)	93 (5.5) #	2001 (4.4)	2193 (5.0)	2366 (5.1)
Renal disease (%)	67 (3.5)	54 (4.3)	69 (4.0)	725 (1.6)	848 (1.9)	1026 (2.2) *
Revascularization (%)	125 (6.5)	120 (9.6) *	208 (12.2) *#	3545 (7.8)	4142 (9.4) *	5179 (11.1) *#
Stroke (%)	127 (6.6)	68 (5.4)	154 (9.0) #	2040 (4.5)	2395 (5.4) *	2730 (5.8) *
LVEF ^3^, %	53.7 ± 10.7	53.9 ± 10.7	52.7 ± 10.1 *#	54.7 ± 10.2	54.4 ± 10.4 *	54.6 ± 10.1

^1^ cardiovascular disease, ^2^ coronary artery disease; ^3^ LVEF, left ventricular ejection fraction. Continuous variable: mean (SD), median (interquartile range); categorical variable: n (%). * *p* < 0.05 compared with pre-outbreak, # *p* < 0.05 compared with outbreak. Number of risk factors was defined as the number of CVD risk factors.

**Table 2:** Patterns of reperfusion strategy and outcomes.

	Hubei			Non-Hubei		
	Pre-Outbreak (*n* = 1913)	Outbreak (*n* = 1253)	Post-Outbreak (*n* = 1705)	Pre-Outbreak (*n* = 45,647)	Outbreak (*n* = 44,008)	Post-Outbreak (*n* = 46,849)
Patient arrival (%)						
EMS	160 (8.4)	131 (10.5)	162 (9.5)	5624 (12.3)	5376 (12.2)	5840 (12.5)
Transfer	616 (32.2)	393 (31.4)	565 (33.1)	13,121 (28.7)	12,684 (28.8)	13,344 (28.5)
Walk-in	1098 (57.4)	715 (57.1)	956 (56.1)	26,071 (57.1)	25,263 (57.4)	26,838 (57.3)
In-hospital onset	39 (2.0)	14 (1.1)	22 (1.3)	831 (1.8)	685 (1.6)	827 (1.8)
Heart rate, bpm	77.6 ± 19.9	78.3 ± 19.1	77.4 ± 19.2	77.7 ± 19.6	77.9 ± 19.4 *	77.0 ± 19.5 *#
Systolic blood pressure, mmHg	132.7 ± 27.0	131.5 ± 26.4	130.8 ± 26.9	133.2 ± 27.2	133.7 ± 27.4 *	131.8±26.9 *#
Patient delay, h	2.1 (1.0–5.9)	2.8 (1.3–6.1) *	2.2 (1.0–5.5) #	2.1 (1.0–5.0)	2.3 (1.1–5.3) *	2.2 (1.0–5.1) *#
Patient delay <12 h (%)	1679 (87.8)	1091 (87.1)	1498 (87.9)	41,560 (91.0)	39,779 (90.4) *	42,542 (90.8) #
Loading drugs						
Aspirin (%)	1725 (90.2)	1131 (90.3)	1531 (89.8)	41,078 (90.0)	39,712 (90.2)	42,118 (89.9)
Clopidogrel (%)	448 (23.4)	383 (30.6) *	422 (24.8) #	17,068 (37.4)	16,928 (38.5) *	16,531 (35.3) *#
Ticagrelor (%)	1278 (66.8)	758 (60.5) *	1125 (66.0) #	24,127 (52.9)	23,218 (52.8)	25,954 (55.4) *#
Reperfusion						
Primary PCI (%)	1447 (75.6)	714 (57.0) *	1256 (73.7) #	33,925 (74.3)	31,940 (72.6) *	35,577(75.9) *#
Thrombolysis (%)	198 (10.4)	300 (23.9) *	185 (10.9) #	6621 (14.5)	7351 (16.7) *	7123 (15.2) *#
system delay (fibrinolysis), min	37.5 (25.0–66.8)	50.0 (30.0–96.5) *	47.0 (28.0–93.3) *	35.0 (25.0–62.0)	35.0 (25.0–65.0)	35.0 (25.0–61.0)
system delay (primary PCI), min	94.0 (69.0–165.8)	130.0 (85.0–221.0) *	106.0 (74.0–189.0) *#	93.0 (69.0–155.0)	99.0 (73.0–163.0) *	96.0 (71.0–158.0) *#
Timely reperfusion (%)	698 (62.2)	310 (41.7) *	599 (55.8) *#	16,040(62.6)	15,977 (59.9) *	19,011 (62.8) #
In-hospital mortality (%)	70 (3.7)	76 (6.1) *	83 (4.9)	1553 (3.4)	1682 (3.8) *	1648 (3.5) #

Continuous variable: mean (SD), median (Q1–Q3); categorical variable: *n* (%). PCI: percutaneous coronary intervention, patient delay: symptom-to-first medical contact, system delay (primary PCI): first medical contact-to-wire crossing time, system delay (fibrinolysis): First medical contact-to-needle. * *p* < 0.05 compared with pre-outbreak, # *p* < 0.05 compared with outbreak.

**Table 3:** Relationship between prevalence of COVID-19 and changes in STEMI management and prognosis in China during the outbreak and post-outbreak periods.

	Outbreak		Post-Outbreak	
	β Coefficient * (95 %CI)	*p* Value	β Coefficient * (95 %CI)	*p* Value
Admissions for STEMI (%)	−2.75 (−5.08~−0.42)	0.02	−1.45 (−3.75~0.85)	0.21
Patient delay (h)	0.05 (0.02~0.08)	0.001	0.02 (−0.04~0.08)	0.59
system delay (primary PCI) (min)	2.85 (−1.12~6.81)	0.15	2.20 (−4.28~8.68)	0.49
system delay (fibrinolysis) (min)	1.13 (−2.02~4.28)	0.28	1·01 (−1.59~3.60)	0.43
Proportion of primary PCI (%)	−1.31 (−2.28~−0.34)	0.01	−0.35 (−1.46~0.75)	0.52
Proportion of thrombolysis (%)	0.81 (−0.07~1.69)	0.07	0.07 (−1.37~1.52)	0.92
Proportion of timely reperfusion (%)	−1.39 (−2.43~−0.35)	0.01	−1.15 (−3·90~1.59)	0.39
In-hospital mortality (%)	0.13 (−0.03~0.30)	0.10	0.10 (−0.12~0.32)	0.37

Adjusted for average age, gross domestic product and population density for 2019, number of chest pain centers and spatial distance from a destination province to Hubei of each province. * β represents the coefficient of multiple linear regression.

## Data Availability

Anonymized data will be available through a formal application process which will be reviewed by the Data Management Committee of the CCA Database—Chest Pain Center.

## Supplementary Materials

The following supporting information can be downloaded at: https://www.mdpi.com/article/10.3390/jcm11247290/s1, Figure S1: Study profile; Figure S2: Changes of patient and system delay; Figure S3: Admission of STEMI cases in different periods; Table S1: National baseline characteristics; Table S2: National reperfusion strategies and in-hospital outcomes.
