# Supplementary material for: The Peripandemic Impact of the First Wave of the COVID-19 Pandemic on Management and Prognosis of ST-Segment Elevation Myocardial Infarction in China
Source: J Clin Med. 2022 Dec 8;11(24):7290. doi: 10.3390/jcm11247290 (PMC9784305; doi:10.3390/jcm11247290)
Supplement: Supplementary file 1 [file jcm-11-07290-s001.zip › jcm-2037196-supplementary.pdf]

Table S1: National baseline characteristics.

	Pre-outbreak (*n*=47560)	Outbreak (*n*=45261)	Post-outbreak (*n*=48554)
**Demographic**			
Age (years)	62.3±13.0	61.9±12.8*	61.8±13.0*
Women (%)	11292 (23.7)	10014 (22.1) *	11074 (22.8) *#
**CVD risk factors**			
Hypertension (%)	16883 (35.5)	17483 (38.6)	19861 (40.9)
Diabetes (%)	6736 (14.2)	7511 (16.6) *	8833 (18.2) *
Dyslipidemia (%)	6816 (14.3)	7409 (16.4) *	8752 (18.0) *#
Smoking (%)	12928 (27.2)	13864 (30.6) *	16131 (33.2) *
Family history of CAD (%)	1268 (2.7)	1275 (2.8)	1376 (2.8)
**Number of risk factors (%)**			
None	8056 (16.9)	8249 (18.2) *	9383 (19.3) *
1	12047 (25.3)	12839 (28.4)	14518 (29.9)
2	7096 (14.9)	7606 (16.8)	8839 (18.2)
≥3	6639 (14.0)	7397 (16.3)	8648 (17.8)
**Medical history**			
Coronary heart disease (%)	13085 (27.5)	14186 (31.3) *	16960 (34.9) *#
Chronic heart failure (%)	2115 (4.4)	2284 (5.0)	2459 (5.1)
Chronic kidney disease (%)	792 (1.7)	902 (2.0)	1095 (2.3)
History of revascularization (%)	3670 (7.7)	4262 (9.4) *	5387 (11.1) *#
Stroke (%)	2167 (4.6)	2463 (5.4) *	2884 (5.9) *
**LVEF (%)**	54.7±10.2	54.4±10.5*	54.6±10.1

Continuous variable: Mean ± SD, Median (Q1-Q3); Categorical variable: n (%)

CAD, coronary artery disease; LVEF, left ventricular ejection fraction

* $p<0.05$ compared with pre-outbreak, #$p<0.05$ compared with outbreak.

Table S2: National reperfusion strategies and in-hospital outcomes.

	**Pre-outbreak** (*n*=47560)	**Outbreak** (*n*=45261)	**Post-outbreak** (*n*=48554)
**Pattern of patient arrival (%)**			
EMS	5784 (12.2)	5507 (12.2) *	6002 (12.4)
Transfer	13737 (28.9)	13077 (28.9)	13909 (28.6)
Walk-in	27169 (57.1)	25978 (57.4)	27794 (57.2)
In-hospital onset	870 (1.8)	699 (1.5)	849 (1.7)
HR, bpm	77.7±19.6	77.9±19.4*	77.1±19.4*#
SBP, mmHg	133.1±27.2	133.7±27.4*	131.8±26.9*#
Patient delay, h	2.1 (1.0-5.0)	2.3 (1.1-5.3) *	2.2 (1.0-5.1) *#
Patient delay <12h (%)	43239 (90.9)	40870 (90.3) *	44040 (90.7)
**Loading drugs**			
Aspirin (%)	42803 (90.0)	40843 (90.2)	43649 (89.9)
Clopidogrel (%)	17516 (36.8)	17311 (38.2) *	16953 (34.9) *#
Ticagrelor (%)	25405 (53.4)	23976 (53.0)	27079 (55.8) *#
**Reperfusion therapies**			
Primary PCI (%)	35372 (74.4)	32654 (72.1) *	36833 (75.9) *#
Thrombolysis (%)	6819 (14.3)	7651 (16.9) *	7308 (15.1) *#
System delay (fibrinolysis), min	35.0 (25.0-62.0)	36.0 (25.0-66.0)	35.0 (25.0-62.0)
System delay (primary PCI), min	93.0 (69.0-156.0)	100.0 (73.0-164.0) *	96.0 (71.0-159.0) *#
Timely reperfusion (%)	16738 (62.6)	16287 (59.4) *	19610 (62.6) #
**In-hospital mortality (%)**	1623 (3.4)	1758 (3.9) *	1731 (3.6) #

Continuous variable: Mean ± SD, Median (Q1-Q3); Categorical variable: n (%)

S-to-FMC: Symptom-to-First Medical Contact, System delay: First Medical Contact-to-Wire crossing time, FMC-to-N: First Medical Contact-to-Needle time, PCI: percutaneous coronary intervention.

* p<0.05 compared with pre-outbreak, #p<0.05 compared with outbreak.

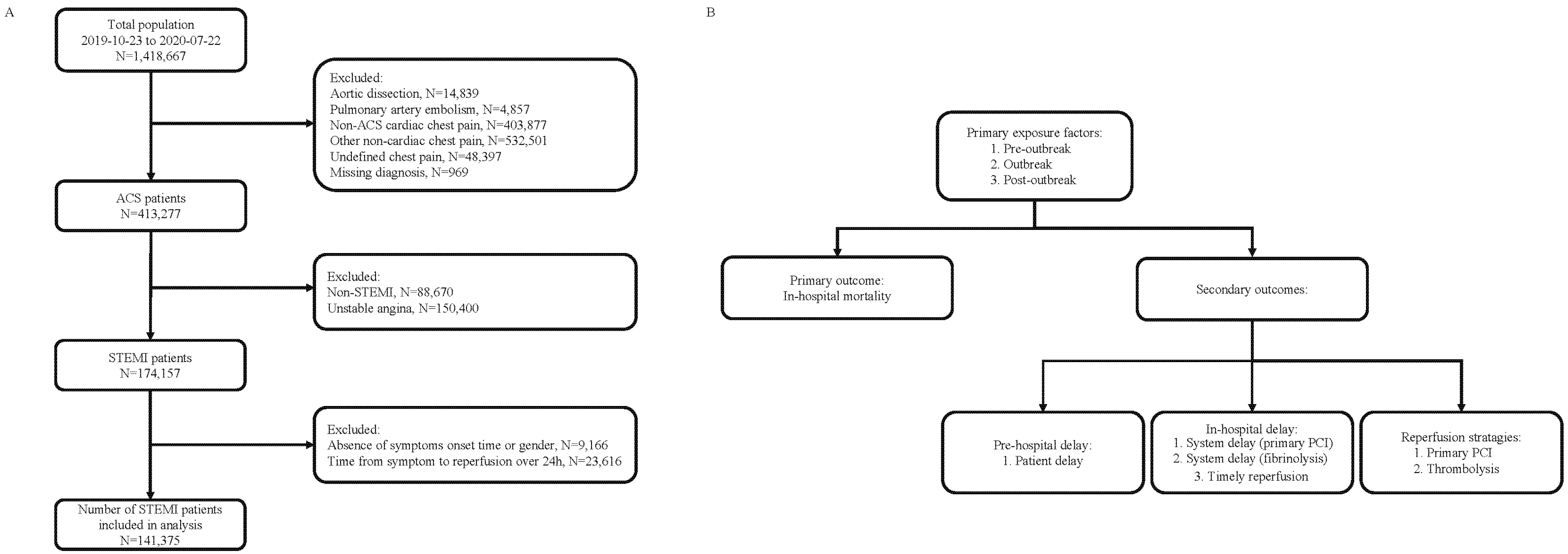

Figure S1: Study profile. Flow chart of screening study population (a) and main exposure and outcome measures in this study (b). ACS, acute coronary syndrome; STEMI, ST-segment elevation myocardial infarction, PCI, percutaneous coronary intervention.

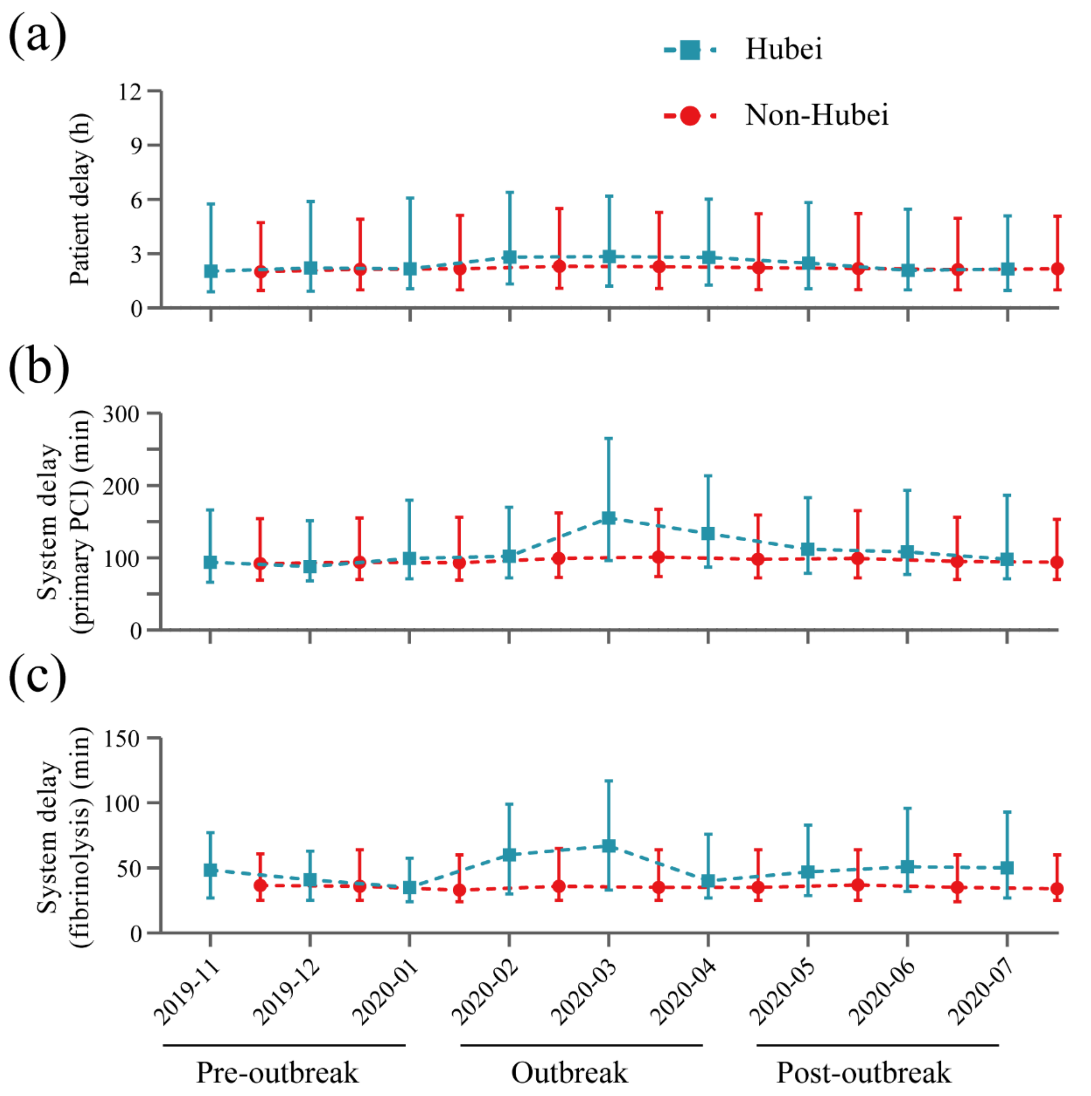

Figure S2. Changes of pre-hospital and in-hospital delay. Times of symptom to first medical contact (patient delay) (a), System delay for primary percutaneous coronary intervention (b), and system delay for thrombolysis (c). Data are presented as median and interquartile range.

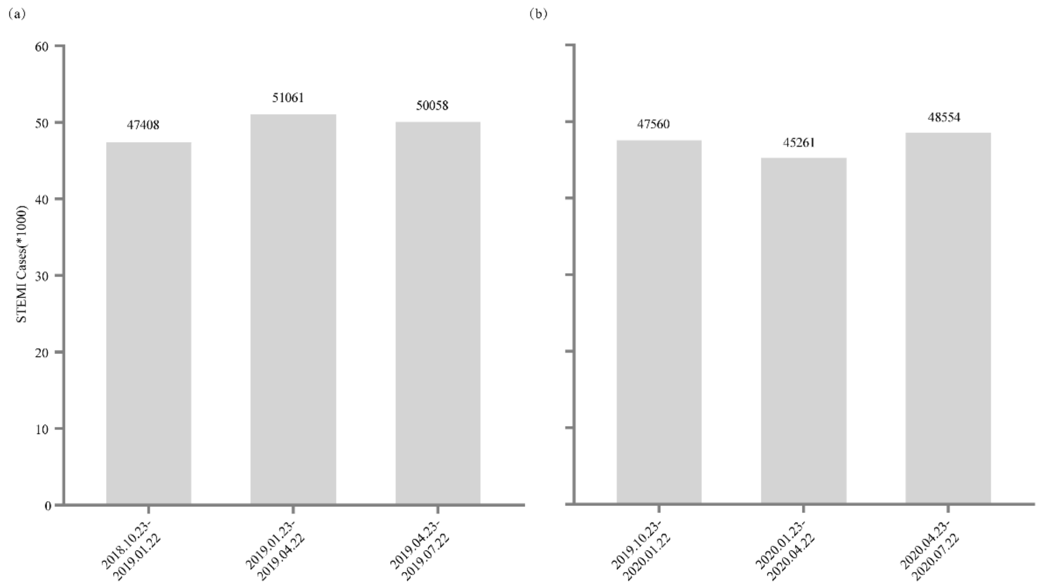

Figure S3: Admission of STEMI cases in different periods. Admission of ST-segment elevation myocardial infarction (STEMI) in different periods in 2018-2019 (a) and 2019-2020 (b).
